# Bile Acid and Cholesterol Metabolism in Atherosclerotic Cardiovascular Disease and Therapy

**Published:** 2020-12-30

**Authors:** John Y. L. Chiang, Jessica M. Ferrell, Yue Wu, Shannon Boehme

**Affiliations:** 1 Department of Integrative Medical Sciences, Northeast Ohio Medical University, Rootstown, OH, USA; 2 Department of Cardiology, First Affiliated Hospital of Xi’an Jiaotong University, Xi’an, China

**Keywords:** Atherosclerosis, Bile acid receptors, Bile acid synthesis, Cholesterol, Lipoprotein

## Abstract

Dysregulation of lipid metabolism is a major factor contributing to atherosclerotic cardiovascular disease (ACVD), which is the number one cause of death in western countries. The liver plays a central role in maintaining whole body cholesterol homeostasis via catabolism of cholesterol to bile acids, as well as biliary cholesterol excretion. The liver synthesizes lipoproteins that transport dietary cholesterol and fats to muscle and adipose tissue, directs reverse cholesterol transport of excess cholesterol from extrahepatic tissues and macrophages to the liver to convert to bile acids, and thus, protects against metabolism-related nonalcoholic fatty liver disease (NAFLD) and ACVD. Liver fibrosis/nonalcoholic steatohepatitis increases the risk and prevalence of cardiovascular disease morbidity and mortality. Bile acids are signaling molecules and metabolic regulators that activate farnesoid X receptor and G protein-coupled bile acid receptor-1 to regulate lipid, glucose, and energy metabolism. The bidirectional regulation of bile acids and the gut microbiota determine the rate of bile acid synthesis, the bile acid pool size, and the composition of the circulating bile acid pool. The liver-intestine-heart axis regulates lipid metabolism, inflammation, and the pathogenesis of metabolic diseases such as ACVD, NAFLD, diabetes, and obesity. This review focuses on the roles of liver-to-intestine, liver-to-heart and intestine-to-heart axes in cholesterol, lipoprotein, and bile acid metabolism; signaling in heart health and ACVD; and drug therapies for atherosclerosis.

## Introduction

Nonalcoholic fatty liver disease (NAFLD) is rapidly increasing worldwide and is an independent risk factor for atherosclerotic cardiovascular disease (ACVD).^[1–4]^ NAFLD can progress to nonalcoholic steatohepatitis (NASH) with macrovascular ballooning, macrophage infiltration, and inflammation and fibrosis of the liver. A recent study has shown that NASH fibrosis can predict ACVD events and that there is a strong association between NASH fibrosis and ACVD morbidity and mortality.^[1]^ ACVD and NAFLD are the manifestations of metabolic syndrome in the heart and liver, respectively. Metabolic syndrome is a collection of five abnormal symptoms: hypertension, hyperglycemia, hypertriglyceridemia, insulin resistance, and obesity.^[5]^

Diabetic and obese persons have a higher risk of NASH and ACVD than nondiabetic and nonobese persons.^[6]^ Both NASH and ACVD are inflammatory diseases having causative factors in common, such as insulin resistance, hyperglycemia, dyslipidemia, and systemic inflammation.^[1,6–8]^ Elevated serum low-density lipoprotein (LDL) cholesterol is a major risk factor for atherosclerosis. Accumulation of oxidized lipids in the intima of blood vessels causes thickening of the arterial wall, as well as inflammation and injury of the coronary artery. Cholesterol, fats, and other substances accumulate and form plaques in the aorta; this restricts blood flow and reduces elasticity of blood vessels.^[7]^

The liver plays a central role in lipid, glucose, and energy metabolism by absorbing dietary fats and cholesterol, synthesizing fatty acids and cholesterol from acetyl-CoA derived from glucose and free fatty acids (FFAs), and distributing fats and cholesterol in lipoproteins to other tissues and organs for energy metabolism.^[9,10]^ Impairment of lipid homeostasis by high fat diet, insulin resistance, and genetic factors results in dyslipidemia, in which increased serum triglycerides (TGs) and cholesterol are linked to the pathogenesis of ACVD and NASH in humans.^[8]^ It has been suggested that “atherosclerosis is a liver disease of the heart” emphasizing the critical role of the liver in producing lipids and lipoproteins to maintain heart health and prevent diseases.^[10]^ This review focuses on the role of the liver-intestine-heart axis in cholesterol and bile acid metabolism and in the pathogenesis of ACVD. Most of the references cited in this review have been published in the last 10 years. Some historical and landmark references have also been cited.

## Cholesterol Metabolism and Homeostasis

Cholesterol is an important component of biological membranes and serves as a precursor of steroids, sex hormones, and bile acids. The liver obtains cholesterol through three cholesterol input mechanisms: dietary absorption, LDL receptor (LDLR)-mediated uptake, and *de novo* synthesis.^[11]^ The liver catabolizes cholesterol to bile acids, which facilitates biliary cholesterol secretion as the predominant cholesterol output mechanism. A small amount of cholesterol is used for cell membrane and steroid hormone synthesis. Cholesterol input and output need to be balanced to maintain whole body cholesterol homeostasis;^[12]^ hence, alteration of cholesterol homeostasis contributes to the pathogenesis of NAFLD and ACVD. A brief description of cholesterol synthesis, lipoprotein metabolism, and bile acid synthesis are presented below.

## *De novo* Cholesterol Synthesis

All tissues and organs in our body can synthesize cholesterol. Cholesterol synthesis in the liver accounts for about 50% of the total cholesterol synthesized daily. In the postprandial state, excess glucose and fatty acids are oxidized to generate acetyl-CoA for the synthesis of cholesterol and fatty acids. In the *de novo* cholesterol synthesis pathway, β-hydroxy-β-methylglutaryl-CoA (HMG-CoA) reductase is the rate-limiting enzyme for cholesterol synthesis. A series of enzymatic reactions convert mevalonate to farnesyl-pyrophosphate, squalene, lanosterol, and finally cholesterol. Cholesterol is a precursor to steroid hormones, vitamin D, and bile acids. *De novo* cholesterol synthesis is regulated by the intracellular levels of cholesterol/oxysterols, which regulate the maturation of sterol regulatory element binding protein-2 (SREBP-2), an important transcription factor that regulates the cholesterol synthesis pathway.^[13]^ When intracellular cholesterol levels are high, the SREBP-2 precursor forms a complex with insulin-induced gene and SREBP cleavage-activating protein (SCAP), and the complex is retained in the endoplasmic reticulum (ER) membrane.^[14]^ When intracellular cholesterol levels decrease, SCAP escorts the SREBP-2 precursor to the Golgi, where two steroid-sensitive proteases cleave an N-terminal fragment transcription factor that is subsequently translocated to the nuclei to activate transcription of its target genes, including LDLR and a number of key genes involved in *de novo* cholesterol synthesis.^[15]^ Activated SREBP-2 binds to the steroid response elements located in the promoters of cholesterol synthesis genes and activates gene transcription. Oxysterols are endogenous ligands of liver X receptor (LXR),^[16,17]^ which plays an important role in the regulation of lipogenic pathways including cholesterol and fatty acid synthesis and transport. Activation of LXR by oxysterols promotes the pathogenesis of atherosclerosis.^[18]^ In contrast, activation of LXR in macrophages protects against atherosclerosis.^[19]^

## Lipoprotein Metabolism

Lipoproteins contain a hydrophobic core, consisting of TGs and cholesterol esters (CEs), and an outer layer, consisting of phospholipids, free cholesterol (C) and apolipoproteins.^[9]^ The major lipoproteins are chylomicrons (CMs), very LDL (VLDL), LDL, and high-density lipoprotein (HDL). These lipoproteins contain several classes of apolipoproteins: ApoA, ApoB, ApoC, ApoD, and ApoE, which serve as structural proteins and effectors or ligands for lipoprotein receptors. These lipoproteins contain different amounts of TGs and cholesterol and transport them to other organs and tissues. Dietary lipids are the major source of fats, phospholipids, and cholesterol in humans. Bile acids released from the gallbladder in the postprandial state emulsify fats to form lipid micelles, which are absorbed in the intestine and transferred to the liver for distribution to other organs and tissues.

## Cholesterol Transport from Liver to Other Tissues

**Figure 1:**
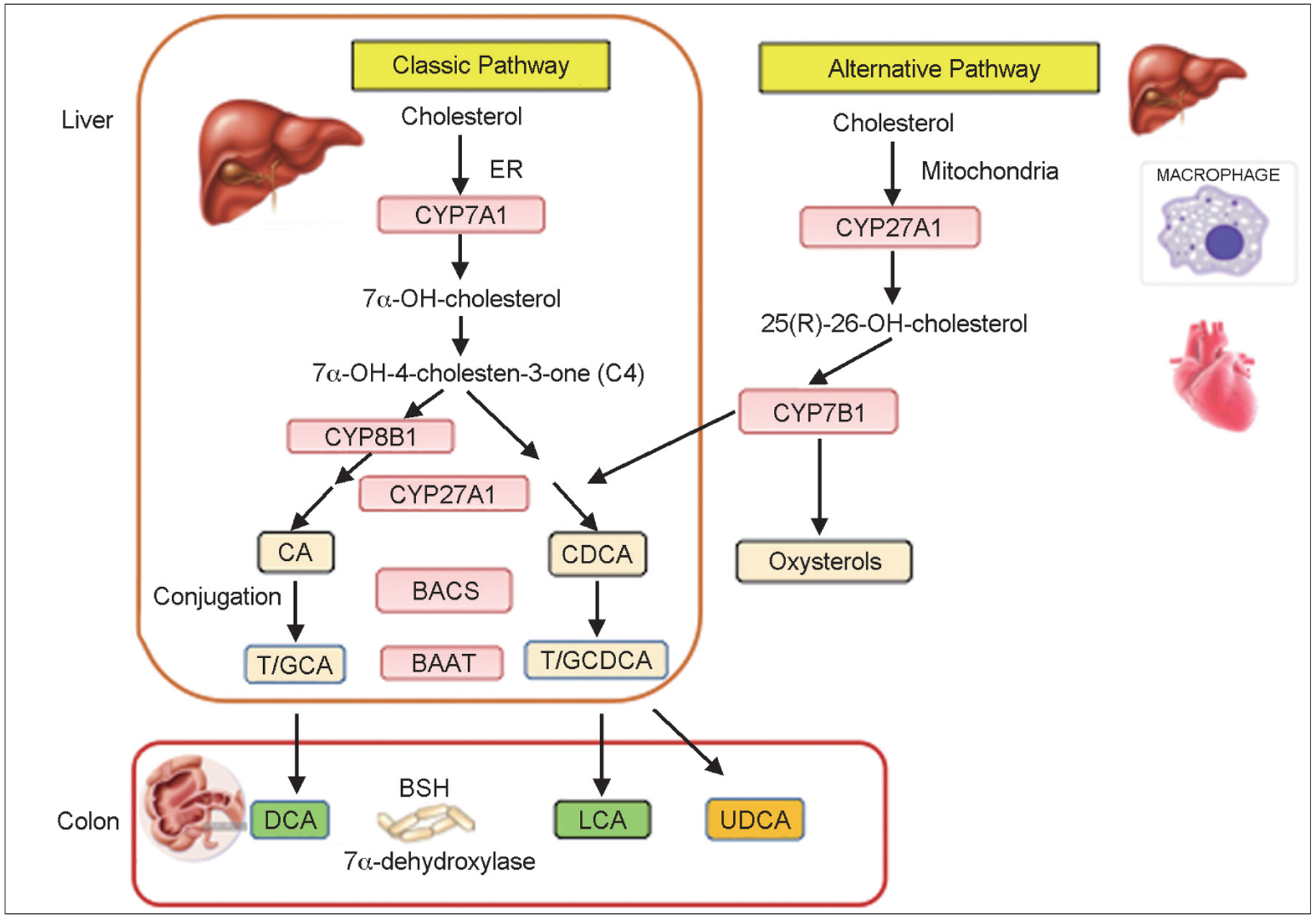
Cholesterol and lipoprotein metabolism. (1) Cholesterol transport from the liver to peripheral tissues; (2) Reverse cholesterol transport from peripheral tissues and macrophages to the liver. ER: endoplasmic reticulum, CYP7A1: cholesterol 7-hydroxylase, CYP8B1: sterol 12-hydroxylase, CYP27A1: sterol 27-hydroxylase, CA: cholic acid, BACS: bile acid CoA synthase, CDCA: chenodeoxycholic acid, DCA: deoxycholic acid, LCA: lithocholic acid, UDCA: ursodeoxycholic acid, CYP7B1: oxysterol 7-hydroxylase

The liver synthesizes lipoproteins and assembles VLDL for the transport of TGs to extrahepatic tissues for energy metabolism^[9]^ [Figure 1]. In the postprandial state, dietary TGs transported to the liver by CMs are hydrolyzed to FFAs and glycerol for energy metabolism. CEs are hydrolyzed to free cholesterol by cholesteryl esterase (CES) for bile acid synthesis. Excess FFAs are re-esterified to glycerol to form TGs and free cholesterol is re-esterified to CE by acyl-CoA:cholesterol acyltransferase 2 for storage and transport to other tissues. Liver microsomal TG transfer protein transfers TGs to ApoB100 (B-100) in the ER to form nascent VLDL, which acquires ApoE and ApoCII from HDL via cholesteryl ester transfer protein (CETP) to form mature VLDL for secretion into blood circulation. On the surface of the blood capillary wall of muscle and adipose tissue, lipoprotein lipase is activated by ApoCII carried by VLDL to hydrolyze TGs to FFAs and glycerol for energy metabolism. VLDL releases some TGs to reduce its size and increase its density to form LDL, which is bound by LDLR, an ApoB100 receptor.

In the liver and other tissues, LDLRs bind ApoB100 carried by LDL to deliver CEs to cells via receptor-mediated endocytosis.^[20]^ Free cholesterol is sorted to the ER and Golgi membranes and is redistributed to intracellular and plasma membranes. Mutations of the LDLR gene impair LDL binding and internalization, as well as lysosomal hydrolysis, recycling and secretion, and cause hypercholesterolemia and severe familial hypercholesterolemia (FH).^[21]^ Patients with FH have very high serum LDL cholesterol (>500 mg/dl) and premature ACVD. More recent studies have identified a natural LDLR inhibitor, proprotein convertase subtilisin kexin type 9 (PCSK9), which regulates the LDLR secretory pathway by stimulating degradation of LDLRs.^[22]^ Mutations in the *PCSK9* gene impair LDLR endocytosis in FH.^[23,24]^ Hepatic PCSK9 expression is regulated by insulin and SREBP-1C.^[25]^

## Reverse Cholesterol Transport

The transport of cholesterol from peripheral tissues and macrophages to the liver for conversion to bile acids is called reverse cholesterol transport (RCT). RCT is the major route for removing excess cholesterol and oxysterols from macrophages to prevent foam-cell formation in the aortic wall and protect against atherosclerosis.^[8,26,27]^ ATP-binding cassette transporter A1 (ABCA1; synthesized in liver and intestine) and ATP-binding cassette transporter G1 (ABCG1) efflux cholesterol and phospholipids to ApoAI to form nascent HDL [Figure 1]. CETP catalyzes the exchange of TGs for CEs and transfers ApoCII and ApoE from VLDL to nascent HDL to form mature HDL.^[28,29]^ In hepatocytes, HDL binds to scavenger receptor-B1 (SR-B1) to deliver TGs and CEs to the liver^[9]^ [Figure 1]. Hepatic lipase on the surface of the hepatic blood capillary wall hydrolyzes TGs to FFAs to deliver FFAs to hepatocytes.^[30]^ In hepatocytes, CE is hydrolyzed to free cholesterol, which is catabolized to bile acids, and this completes the RCT process. Stimulating RCT from macrophages to liver accelerates conversion of cholesterol to bile acids and protects against atherosclerosis.

## Bile Acid Synthesis

**Figure 2:**
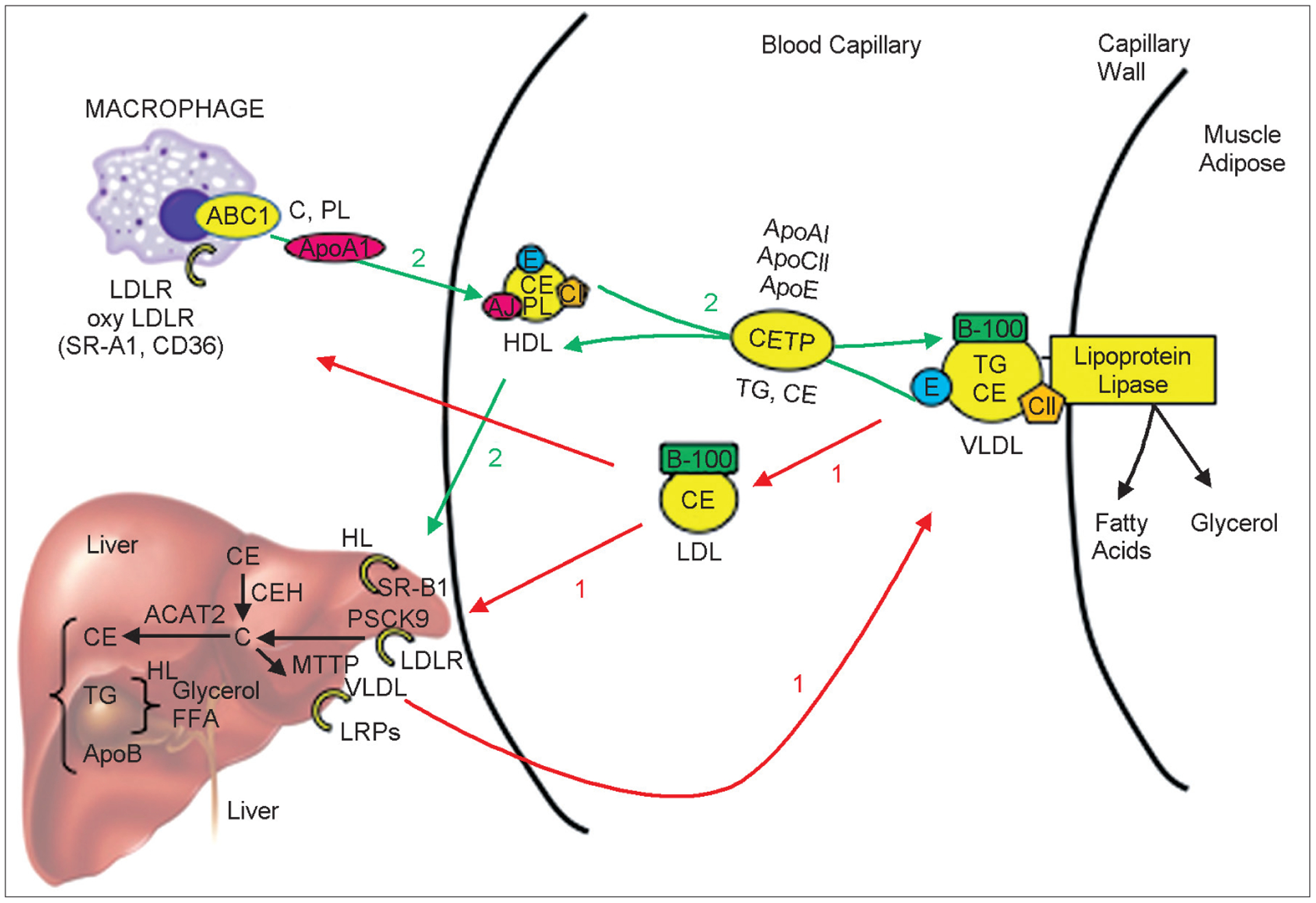
Bile acid synthesis in the liver, heart and other tissues, and biotransformation in the intestine. LDLR: LDL receptor, SR-A1: scavenger receptor A1, CE: Cholesterol esters, ACAT2: acyl-CoA: cholesterol acyltransferase 2, C: cholesterol, MTTP: microsomal triglyceride transfer protein, HL: Hepatic lipase, SR-B1: scavenger receptor B1, PCSK9, proprotein convertase subtilisin kexin type 9, LDLR: LDL receptor, VLDL: very low-density lipoprotein, LRP: LDL receptor related protein, HL: Hepatic lipase, TG: triglycerides, FFA: free fatty acids, CETP: cholesteryl ester transfer protein

The liver exclusively contains all the enzymes required for catabolism of cholesterol to bile acids, which is the predominant use of cholesterol in the body. Bile acid synthesis involves 17 enzymes located in microsomes, cytosol, mitochondria, and peroxisomes.^[31–33]^ Here, only the regulatory cytochrome P450 (CYP) enzymes in bile acid synthesis are described. In humans, cholic acid (CA) and chenodeoxycholic acid (CDCA) are the two primary bile acids synthesized in the liver [Figure 2]. Cholesterol 7α-hydroxylase (CYP7A1) catalyzes the first and rate-limiting step in the classic bile acid synthetic pathway to synthesize 7α-hydroxycholesterol, which is converted to 7α-hydroxy-4-cholesten-3-one (C4). Serum C4 levels are currently used as a surrogate marker for the rate of bile acid synthesis. Sterol 12α-hydroxylase (CYP8B1) is required for hydroxylation at the C-12 position leading to synthesis of CA; in the absence of this step, CDCA is produced. Mitochondrial sterol 27-hydroxylase (CYP27A1) oxidizes the steroid sidechain of intermediates leading to cleavage of a 3C unit to produce C24 bile acids. Bile acids recycled to the liver and bile acid-CoA synthesized in the liver are immediately conjugated to the amino acid taurine (T) or glycine (G) by bile acid:amino acid transferase and bile acid:CoA synthase, respectively, to form T-or G-conjugated bile acids, which are secreted into bile. The alternative bile acid synthesis pathway is initiated by CYP27A1 to generate 27-hydroxycholesterol from cholesterol. The nonspecific oxysterol 7α-hydroxylase (CYP7B1) catalyzes 7α-hydroxylation of 27-hydroxycholesterol. CYP27A1 is highly expressed in macrophages to generate 27-hydroxycholesterol, which is 7α-hydroxylated by CYP7B1.^[34]^ 27-Hydroxycholesterol is an endogenous LXR agonist in cholesterol-loaded macrophages.^[35]^ Cholesterol-loading activates LXR to induce ABCA1 and ABCG1 in macrophages to efflux cholesterol and oxysterols [Figure 2]. It has been suggested that the transport of 27-hydroxycholesterol from macrophages to the liver for bile acid synthesis is a RCT process to protect against atherosclerosis.^[36]^ CYP27A1 and CYP7B1 are expressed in many extrahepatic tissues, such as the heart, brain, and kidney, and play a key role in the regulation of oxysterol synthesis and steroid hormone synthesis in the adrenal glands.^[34]^

## Transformation of Bile Acids in the Gut

The gut-to-liver axis plays a critical role in bile acid synthesis and metabolism.^[37]^ The gut microbiota metabolizes primary bile acids to secondary bile acids, which in turn control gut bacterial overgrowth. In the postprandial state, the duodenum releases cholecystokinin to stimulate gallbladder contraction and secretion of bile into the gastrointestinal tract for emulsification of fats and nutrients. Most bile acids (~95%) are reabsorbed in the ileum. A small amount of bile acids enter the colon, where gut bacterial bile salt hydrolases (BSHs) de-conjugate T/GCA and T/GCDCA [Figure 2].^[38,39]^ Subsequently, bacterial 7α-dehydroxylase removes a 7-hydroxyl group from CA and CDCA to form the secondary bile acids deoxycholic acid (DCA) and lithocholic acid (LCA), respectively. These secondary bile acids are highly insoluble and toxic and are mostly excreted in the feces. Some DCA is passively reabsorbed in the colon and secreted into the circulating bile acid pool. Most LCA is excreted in feces; a small amount of LCA circulated to the liver is rapidly sulfur-conjugated by sulfotransferases and excreted in urine. In humans, a small amount of CDCA is converted to ursodeoxycholic acid (UDCA) by bacterial 7α/β-hydroxysteroid dehydrogenase. Isomerization of CDCA to UDCA converts the highly hydrophobic CDCA to the highly soluble UDCA. In humans, the bile acid pool consists of the hydrophobic bile acids CA, CDCA, and DCA in an approximate 40:40:20 ratio, and bile acids are conjugated to G and T in a ratio of 3:1. The gut bacterial enzymes BSH and 7α-dehydroxylase play a critical role in the regulation of bile acid synthesis, pool size, and homeostasis. Alteration of bile acid composition and pool size by gut bacteria causes dysbiosis and significantly impacts the host metabolism, as well as pathogenesis of metabolic liver and heart diseases.^[39–43]^

## Enterohepatic Circulation of Bile Acids

**Figure 3:**
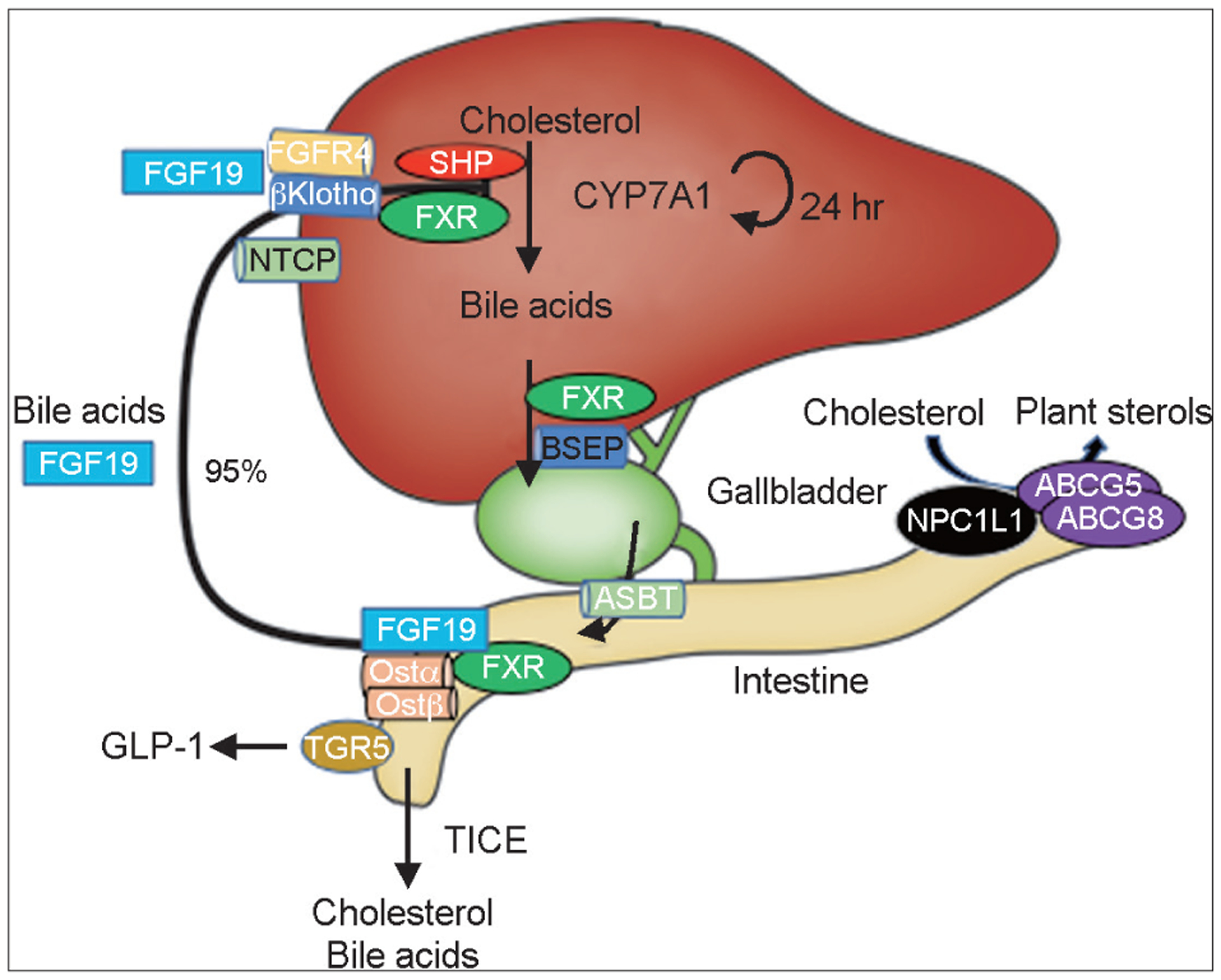
Farnesoid X receptor regulation of enterohepatic circulation of bile acids and transintestinal cholesterol excretion. FGF19: fibroblast growth factor 19, SHP: small heterodimer partner, FXR: farnesoid X receptor, CYP7A1: cholesterol 7-hydroxylase, NTCP: Na+-dependent taurocholate co-transport peptide, BSEP: bile salt export pump, ASBT: apical sodium-dependent bile acid transporter, TGR5: Takeda G protein receptor 5, GLP-1: glucagon-like peptide 1, TICE: transintestinal cholesterol excretion, NPC1L1: Niemann-Pick C1-like protein

In the ileum, conjugated bile acids are reabsorbed along with fats, cholesterol, and lipid-soluble vitamins for delivery to the liver and distribution to other organs and tissues. This enterohepatic circulation (EHC) of bile acids from the intestine to the liver is highly efficient, recovering ~95% of bile acids in the pool to inhibit bile acid synthesis in the liver [Figure 3].^[31,44]^ A small amount (5%) of bile acids lost in feces is replenished by *de novo* synthesis in the liver. The EHC of bile acids involves several bile acid transporters [Figure 3].^[32]^ At the canalicular membrane, bile salt export pump (BSEP, ABCB11) effluxes bile acids to bile, multidrug resistance associated protein (ABCC2, MRP2) effluxes conjugated-bile acids, multi-drug resistant protein 2 (MDR2, ABCB4) effluxes phospholipids and the ABCG5/G8 heterodimer effluxes cholesterol into bile. Bile acids, phospholipids, and cholesterol form mixed micelles in bile to increase cholesterol solubility and reduce bile acid toxicity. Bile acids are reabsorbed into intestinal cells by apical sodium-dependent bile acid transporter (ASBT) located in the brush border membrane and are transported to the basolateral membrane for secretion into portal blood by the heterodimeric organic solute transporters (OSTα and OSTβ). In the apical membrane of enterocytes, the ABCG5/G8 transporter effluxes plant sterols and cholesterol to prevent absorption of plant sterols and limit dietary cholesterol absorbed by Niemann-Pick C1-like protein (NPC1 L1). In hepatocytes, the bile acid transporter Na^+^-dependent taurocholate co-transport peptide (NTCP) located in the sinusoidal membrane absorbs bile acids in exchange for Na^+^. These bile acid transporters may play an important role in protection against cholestasis when bile acids accumulate in hepatocytes. Interrupted EHC of bile acids contributes to metabolic syndrome, cholestatic liver disease, inflammatory bowel disease, diarrhea, and gallstone disease.^[32,45]^

## Transintestinal Cholesterol Excretion

Biliary cholesterol secretion and catabolism of cholesterol to bile acids are the main mechanisms for cholesterol excretion from the body. RCT is the main pathway for removing excess cholesterol from peripheral tissues and macrophages. However, recent studies have implicated a role for the intestine in nonbiliary cholesterol excretion via direct fecal neutral sterol excretion called transintestinal cholesterol excretion [TICE; Figure 3]. In the intestine, NPC1 L1 and ABCG5/G8 are involved in TICE.^[46,47]^ TICE is inducible by PCSK9 inhibitors or HMG-CoA reductase inhibitors (statins).^[48,49]^ In both mice and humans, TICE can be stimulated by a NPC1 L1 inhibitor, ezetimibe, which induces ABCG5/ABCG8 to efflux cholesterol from the intestine to prevent atherosclerosis.^[50,51]^

## Bile Acid-Activated Receptors in Atherosclerosis

Bile acid synthesis and the EHC of bile acids are regulated by the bile acid-activated nuclear receptor farnesoid X receptor (FXR).^[32,52]^ FXR plays a central role in the regulation of glucose, lipid, and energy metabolism.^[53,54]^ FXR is highly expressed in the gastrointestinal tract, acts as a sensor of bile acid levels in hepatocytes and enterocytes, and coordinately regulates transcription of a network of genes in bile acid synthesis, conjugation, and transport. In the liver, activation of FXR by bile acids induces the nuclear receptor small heterodimer partner, which negatively regulates bile acid synthesis by inhibiting *CYP7A1* gene transcription [Figure 3].^[55,56]^ FXR also induces BSEP to stimulate bile acid efflux into bile and inhibits NTCP to suppress bile acid uptake by hepatocytes.^[57]^ In the intestinal ileum, FXR induces an enteroendocrine hormone known as fibroblast growth factor 19 (FGF19) [Figure 3].^[58]^ FGF19 circulates to hepatocytes to activate the FGF receptor 4/β-Klotho complex on the cell membrane, inhibiting *CYP7A1* and *CYP8B1* gene transcription [Figure 3].^[32,58]^

Activation of FXR has been shown to stimulate RCT, increase fecal cholesterol excretion, and reduce pro-inflammatory cytokines to attenuate atherosclerosis.^[30,59–61]^ FXR is expressed in vascular smooth muscle and atherosclerotic blood vessels.^[62]^ Accumulation of bile acids has been shown to induce cardiomyopathy and cardiac function by decreasing expression of peroxisome proliferator-activated receptor γ coactivator 1α (PGC-1α), which is involved in energy metabolism in the heart.^[63]^ It has been reported that activation of FXR contributes to myocardial ischemia-reperfusion injury.^[64]^ Deficiency of FXR impairs bile acid synthesis and increases serum bile acids, cholesterol, TGs, and pro-atherogenic lipoprotein profile in mice.^[65]^ However, activation of FXR reduces ApoAI and ApoCIII expression; thus, it may reduce serum HDL and TGs in humans.^[66,67]^

The secondary bile acids LCA and DCA activate G-protein coupled bile acid receptor-1 (Gpbar-1, also known as TGR5) in enteroendocrine L cells to stimulate secretion of glucagon-like peptide 1 (GLP-1), which stimulates insulin secretion in the pancreas to improve insulin sensitivity [Figure 3].^[32,68,69]^ TGR5 is widely expressed in most tissues, including the heart. Activation of TGR5 has been shown to improve myocardial function,^[70]^ induce nitric oxide production and reduce monocyte adhesion in vascular endothelial cells,^[71]^ stimulate smooth muscle relaxation,^[72]^ and reduce macrophage inflammation and atherosclerosis.^[73,74]^ Activation of both FXR and TGR5 protects mice against atherosclerosis.^[75]^

## Gut Microbiota and Atherosclerosis

**Figure 4:**
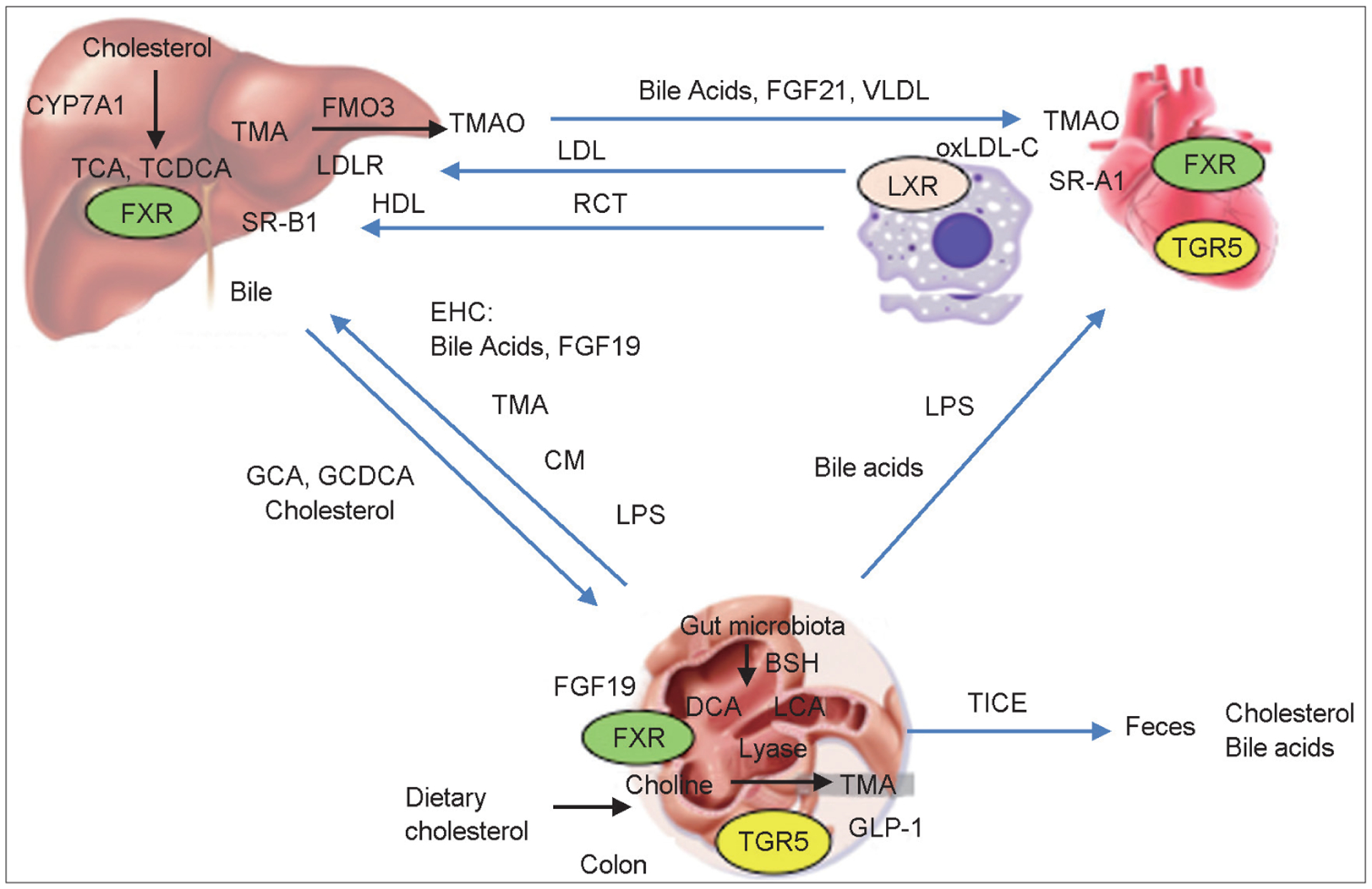
Interactions of the liver, heart, and intestine in lipid metabolism. The liver-to-heart, liver-to-intestine, and intestine-to-heart axes are shown. CYP7A1: cholesterol 7-hydroxylase, TMA: trimethylamine, FXR: farnesoid X receptor, SR-B1: scavenger receptor B1, TMAO: trimethylamine-oxide, VLDL: very low-density lipoprotein, LDL: low-density lipoprotein, RCT: reverse cholesterol transport, TGR5: Takeda G protein receptor 5, EHC: enterohepatic circulation, TMA: trimethylamine, CM: chylomicrons, FGF19: fibroblast growth factor 19, BSH: bile salt hydrolases, DCA: deoxycholic acid, LCA: lithocholic acid, TMA: trimethylamine, GLP-1: glucagon-like peptide 1, TICE: transintestinal cholesterol excretion

The role of the gut microbiota in lipid metabolism and atherosclerosis has been implicated.^[43,76–81]^ Proinflammatory gut bacteria have been shown to increase systemic inflammation and promote atherosclerosis in mice.^[43]^ Secondary bile acids and lipopolysaccharide (LPS) generated by intestinal bacteria are transported to the heart and liver and cause inflammation. The gut bacteria convert choline (derived from phosphatidylcholine) and L-carnitine to trimethylamine (TMA) by the action of TMA lyases [Figure 4]. TMA is circulated to the liver and converted to trimethylamine-oxide (TMAO) by flavin-containing monooxygenase 3 (FMO3). Serum TMAO levels have been linked to increased cardiovascular events.^[82,83]^ TMAO impairs RCT, as indicated by increased macrophage cholesterol and oxLDL, CD36 and SR-A1 receptors, and foam-cell formation, to promote atherosclerosis in a gut microbiota-dependent manner.^[78]^ TMAO supplementation in diet suppresses CYP7A1 and CYP27A1 expression, increases hepatic cholesterol, and decreases the total bile acid pool.^[78]^ TMAO also suppresses expression of intestinal NPC1 L1 and ABCG5/ABCG8, thereby reducing intestinal cholesterol absorption.^[78]^

## Lipid-Lowering Therapies for Treating Atherosclerosis and Familial Hypercholesterolemia

Serum bile acid levels vary in individuals, and increased fasting bile acids and serum C4 are correlated with type 2 diabetes (T2D) and ACVD.^[84]^ Disruption of RCT contributes directly to the accumulation of oxidized LDL and fatty foam cells in the endothelium of the aortic wall and to the pathogenesis of atherosclerosis. A study conducted about 50 years ago reported that interruption of the EHC of bile acids by cholestyramine increased ileal excretion of cholesterol and reduced hypercholesterolemia in humans.^[85]^ Cholestyramine reduces serum cholesterol and LDL-cholesterol in T2D.^[86]^ Cholestyramine reduces the bile acid pool to stimulate bile acid synthesis and increases LDLR gene expression to reduce hepatic cholesterol and LDLR-mediated uptake of cholesterol from blood circulation, thereby reducing hypercholesterolemia.^[87]^ Patients with hyperlipoproteinemia treated with cholestyramine had increased bile acid and TG synthesis, whereas those treated with CDCA had reduced bile acid and TG synthesis.^[88]^ Cholestyramine also reduces FGF19 induced by FXR in intestine, thus derepressing CYP7A1 to stimulate bile acid synthesis.^[89]^ Cholestyramine, colestipol, and second generation bile acid binding resins colesevelam and colestimide have been used to treat hypercholesterolemia, cholesterol gallstone disease, and T2D.^[90–93]^ Cholestyramine significantly reduced atherosclerosis progression in a clinical trial of patients with coronary heart disease.^[94]^ Bile acids may be used to treat atherosclerosis by regulating lipoprotein metabolism. ABCA1, ABCG1, and HDL are potential targets for the treatment of atherosclerosis.^[95]^

Statins are potent lipid-lowering drugs that inhibit HMG-CoA reductase activity in the *de novo* cholesterol synthesis pathway, and thus, have been used successfully to reduce the risk of ACVD events in humans. Although statins alone are not sufficient for treating FH, a combination of statins and cholestyramine has been shown to alter lipoprotein profile and lower LDL cholesterol, as well as to safely treat FH.^[96–98]^ However, statins are not recommended for pregnant women and some patients who are resistant to statins may develop unwanted side effects of muscle pain and liver injury. Drugs designed to increase HDL cholesterol have been further developed for treating FH. Ezetimibe and niacin modestly reduce LDL cholesterol and increase HDL cholesterol. Torcetrapib, a CETP inhibitor, has been shown to effectively increase HDL cholesterol levels and stimulate RCT in mice^[99]^ and humans.^[29,100]^ However, a clinical trial of torcetrapib for atherosclerosis was withdrawn due to high mortality rate.^[101,102]^ As an alternative to statins, PCSK9 inhibitors were developed for reducing LDL cholesterol and ACVD risk.^[103]^ PCSK9 inhibitors have been shown to augment circulating LDLRs to hepatocytes to accelerate clearance of LDL. The FDA recently approved two PCSK9 monoclonal antibodies for treating FH. Statins inhibit the synthesis of oxysterols and reduce the activation of LXR by oxysterols in hepatocytes.^[104]^ Activation of LXR specifically in macrophages reduces inflammation and atherosclerosis. Therefore, drugs specifically targeting macrophages may be designed to treat atherosclerosis.^[105,106]^

Obeticholic acid (OCA) is a potent bile acid derivative that activates FXR to reduce hepatic lipid synthesis and improve glucose and insulin sensitivity in patients with NAFLD.^[37,107–109]^ OCA may also be used to treat atherosclerosis. Activation of FXR represses PCSK9 in human hepatocytes.^[110]^ However, OCA reduces serum ApoAI and HDL cholesterol levels, and may have adverse effects in atherosclerosis.^[8,111,112]^

## Conclusion

The liver plays a central role in lipid metabolism and homeostasis by providing TGs and cholesterol to the heart and other tissues. Catabolism of cholesterol to bile acids and biliary cholesterol secretion are the predominant mechanisms to remove excess cholesterol from the body and protect against atherosclerosis. Bile acid signaling via activating FXR in the gut-to-liver axis plays a key role in the regulation of the EHC of bile acids, as well as bile acid synthesis, composition, and pool size, to maintain bile acid homeostasis and regulate whole body lipid homeostasis.^[41,76,77,113,114]^ The liver, heart, and intestine are linked by lipoproteins, bile acids and gut bacterial metabolites to control bile acid and cholesterol signaling [Figure 4]. The liver-to-heart axis is regulated by normal cholesterol transport to deliver cholesterol to the heart, RCT to transport oxidized cholesterol from macrophages to the liver for catabolism to bile acids, and LDLR-mediated endocytosis to remove excess cholesterol from macrophages and extrahepatic tissues to prevent atherosclerosis. The liver-to-intestine axis is important to bile acid metabolism and to the control of gut bacterial overgrowth to prevent inflammation and injury to the liver and heart. The intestine can directly excrete cholesterol to feces through TICE. The intestine-to-liver axis and intestine-to-heart axis link the gut bacteria metabolite TMA to the liver to form TMAO, which impairs RCT and atherosclerosis. Further study of the impact of the gut microbiota on heart diseases will be important for developing alternate drug therapies to the use of statins for ACVD. Probiotics may be used for ACVD treatment in human patients in the future, as they modify gut microbiota and induce bile acid synthesis and have been shown to protect against NASH and atherosclerosis in mouse models.^[115–118]^
